# Neural Systems Involved When Attending to a Speaker

**DOI:** 10.1093/cercor/bhu325

**Published:** 2015-01-16

**Authors:** Salwa Kamourieh, Rodrigo M. Braga, Robert Leech, Rexford D. Newbould, Paresh Malhotra, Richard J. S. Wise

**Affiliations:** 1 Computational, Cognitive, and Clinical Neuroimaging Laboratory, Division of Brain Sciences, Imperial College London, Hammersmith Hospital Campus, London W12 0NN, UK; 2 Imanova Centre for Imaging Sciences, Hammersmith Hospital Campus, London W12 0NN, UK

**Keywords:** attention, cognitive control, functional mRI, right anterior insula, speech

## Abstract

Remembering what a speaker said depends on attention. During conversational speech, the emphasis is on working memory, but listening to a lecture encourages episodic memory encoding. With simultaneous interference from background speech, the need for auditory vigilance increases. We recreated these context-dependent demands on auditory attention in 2 ways. The first was to require participants to attend to one speaker in either the absence or presence of a distracting background speaker. The second was to alter the task demand, requiring either an immediate or delayed recall of the content of the attended speech. Across 2 fMRI studies, common activated regions associated with segregating attended from unattended speech were the right anterior insula and adjacent frontal operculum (aI/FOp), the left planum temporale, and the precuneus. In contrast, activity in a ventral right frontoparietal system was dependent on both the task demand and the presence of a competing speaker. Additional multivariate analyses identified other domain-general frontoparietal systems, where activity increased during attentive listening but was modulated little by the need for speech stream segregation in the presence of 2 speakers. These results make predictions about impairments in attentive listening in different communicative contexts following focal or diffuse brain pathology.

## Introduction

Listening to a speaker so that what was said is understood and remembered requires attention. The duration of both attention and the time over which the content of what was heard has to be remembered is influenced by context. Thus, taking turns during conversations depends on periods of brief attentive listening, with memory focused principally on what was just said before making a response. In this context, the emphasis is on working memory. In contrast, attendance at a lecture is an hour well spent only if the listener reliably maintains attention over time while encoding details of the semantic content of the lecture as enduring memories.

In addition to the communicative goal, another factor influencing attentive listening is the auditory environment. We often hear speech in social situations, so that the “attended” speech has to be segregated from that of other speakers in the near vicinity. Research on speech-in-speech masking has been mainly directed at the auditory cues that listeners use to overcome the peripheral (energetic, at the level of the cochlea) and central (informational) masking (Brungart 2001). These include spatial information, differences in voice pitch and prosody, and the asynchrony of the onset and offset of speech sounds (Bregman 1990; Darwin and Hukin 2000a; 2000b; Feng and Ratnam 2000; Carlyon 2004; Snyder and Alain 2007; Darwin 2008).

Less research has been directed at the demands made on domain-general systems for attention that contribute to understanding and remembering what a speaker has said in different communicative contexts, and how their function alters in response to the challenge of listening to a speaker in the presence of background speech. The 2 functional neuroimaging studies reported here investigated whole-brain activity as normal participants attended to the verbal message conveyed by a speaker. On different trials, the speaker was talking in the absence or presence of another speaker. In both studies, the “attended” and “competing” speakers were of different sex, offering cues conveyed by pitch and vocal-tract sizes to assist in the segregation of competing speech streams. Spatial information was also included in some trials in one of the studies. Importantly, the task demand was altered between the 2 studies. In one, the participants knew that they would answer questions about what they had heard only after the completion of scanning, requiring the series of sentences to be encoded as episodic memories. In the other, the participants responded to a question immediately after each sentence, thereby placing demands mainly on working memory.

The aim was to investigate the participation of networks involved in domain-general attention and cognitive control as participants listened to speech. The existence of these networks is well established, and they have been demonstrated in a wide range of functional neuroimaging studies. Although it is accepted that they are involved in top-down control across a broad range of task contexts and there is consensus about their anatomical distribution, whether they are truly divisible in terms of functional dissociations and the precise nature of their processing roles is the subject of continuing research on humans. This is based on both functional neuroimaging studies on normal participants and lesion-deficit analyses on patients with focal lesions (examples, from numerous publications of original research and review articles, include: Dosenbach et al. 2007; 2008; Corbetta et al. 2008; Shallice et al. 2008; Vincent et al. 2008; Singh-Curry and Husain 2009; Duncan 2010; Hampshire et al. 2010; Menon and Uddin 2010; Roca et al. 2010; Corbetta and Shulman 2011; Woolgar et al. 2011; Hampshire et al. 2012; Woolgar et al. 2013; Duncan 2013; Aron et al. 2014).

In the 2 studies presented here, we refer to 4 of these networks by anatomical labels. Based on the published literature, cited earlier, these consist of 2 dorsal frontoparietal systems, symmetrically distributed between the hemispheres, a third, more ventral, frontoparietal system that is usually considered to be predominantly right-lateralized, and a fourth that is distributed between dorsal midline frontal cortex and bilateral anterior insular and adjacent frontal opercular cortex. The present studies were designed to investigate activity across these networks during speech comprehension, and differences, if any, of the modulation of this activity by contexts encountered in everyday life. A better understanding of the participation of these networks in communication in the normal brain will inform a common problem encountered by patients with diverse common pathologies, such as stroke, neurodegenerative disease, or traumatic brain injury (TBI). These patients often find that attending to speakers when they are distracted by background speech, or when they have to pay attention over longer periods, is particularly problematic. As a consequence, the additional impairment in registering verbal information will aggravate any deficit in the encoding of verbal information. As attention and cognitive control are potential targets for symptom-modifying pharmacotherapy (for example, Klinkenberg et al. 2011; Robertson 2014), the present study anticipates future investigations of the diseased brain and its potential response to such agents.

## Materials and Methods

### Subjects

Two experiments were performed. One involved 29 healthy participants (11 females, 2 left-handed) with a mean age of 44 years (range 23–71). In the second, there were 25 healthy participants (13 females, all right-handed) with a mean age of 66 years (range 51–83). The subjects were recruited from the community, through personal contacts and advertisements. None had a history of neurological or psychiatric disorders. Although none reported difficulty with hearing, the loudness of the stimuli was adjusted for each participant to a level at which they reported they could hear the stimuli clearly during scanning. All had normal or corrected-to-normal vision. Written consent was obtained from all participants, with prior approval from the North West Thames ethics committee.

### Experiment 1

#### Stimuli

**Figure 1.**
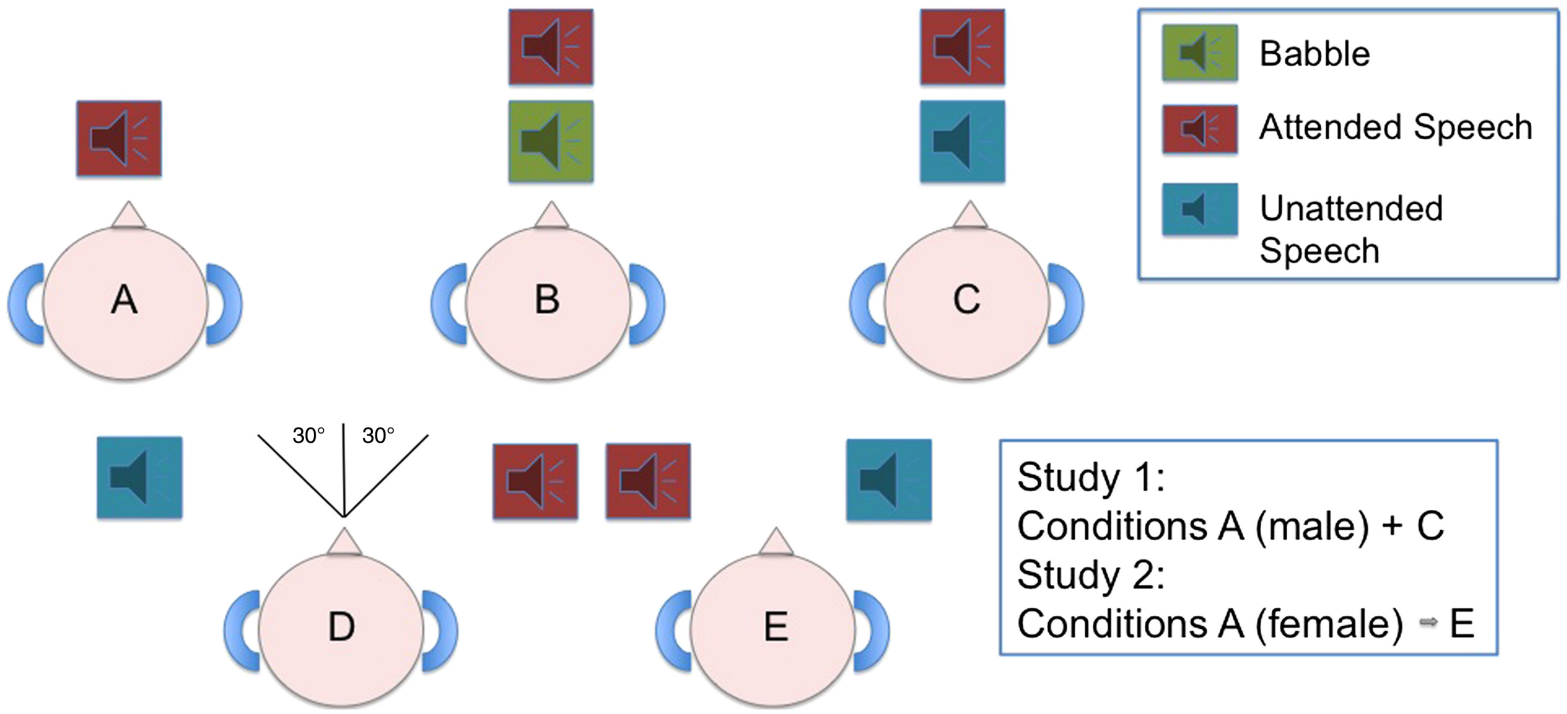
A diagrammatic representation of the auditory conditions heard during Studies 1 and 2. Study 1 included Conditions A + C. Study 2 had all 5 auditory conditions. (A) The attended speaker alone (Study 1 = male, Study 2 = female); (B) background babble delivered with the attended speaker through the same channel (diotic presentation); (C) simultaneous voices of a male and female speaker presented diotically; (D + E) Competing female and male speech, as Condition C, presented dichotically with a spatial cue (either with the female speaker at 30° to the left and the male speaker at 30° to the right, or vice versa).

There were 2 auditory speech conditions, with equal numbers of stimuli in each condition. In the first, the participants heard the voice of a male speaker alone. In the second, they heard the simultaneous voices of a male and female speaker, with the separate voices mixed into the same channel; that is, diotic presentation with no spatial cues (Fig. 1). The participants were informed before the start of scanning that they would be asked questions about what the male speaker had said at the completion of scanning, without knowing in advance what form these questions would take.

All sentences, spoken by the male or female, were recorded in an anechoic chamber and adjusted to 2-s duration, using Sound Studio 2.2.4 (Felt Tip software www.felttip.com). The participants were required to attend to the sentences spoken by the native English male speaker. These sentences were taken from the Speech Intelligibility in Noise (SPIN) test (Kalikow et al. 1977). They have previously been used in an fMRI study on language comprehension (Obleser et al. 2007). Sixty-four sentences were randomly chosen from SPIN. All sentences ended in a noun, and these final nouns were reallocated to produce an equal number of sentences each with a semantically incongruous noun ending. This resulted in a total of 128 sentences. The addition of an incongruous ending to half the sentences spoken by the male speaker was intended to determine whether breeching an anticipated sentence ending modulated activity within the higher-order networks regulating attention and cognitive control during speech comprehension.

The recorded sentences were adjusted using Praat (http://www.fon.hum.uva.nl/praat/) to have the same root-mean-squared average intensity. The sentences spoken by the native English female speaker were recorded and adjusted in the same manner as those spoken by the male speaker. She read aloud sentences from a variety of sources, including subsections of contemporary news stories, Wikipedia, and a children's book. During the diotic presentation of 2 speakers, the male and female sentences were mixed together equally with a 0-dB signal-to-noise ratio. In addition, there were 2 low-level baseline conditions: one with bursts of a continuous pure tone at 400 Hz, without any task demand (Tones) and one with no auditory stimuli (Silence). The 400-Hz tone bursts were adjusted to have the same duration and equivalent root-mean-squared intensity to the sentences.

#### Study Design

The study relied on “sparse sampling” (Hall et al. 1999) during functional image acquisition, so that all stimuli were heard without masking by background scanner noise. For an individual trial, the stimuli were presented during a period of 8 s when there was no data acquisition (and hence no scanner noise). Data were then acquired during the ensuing 2 s. As soon as one epoch of data acquisition was completed, a visual cue, “listen to the male voice”, would appear and remain for 8 s when the sentences were presented. When the pure tones were presented, the visual cue was “listen to the sounds.” During a sixth condition, without the presentation of stimuli or any task demand (Silence), the participants saw the single word “relax.” During each trial, the participants listened through ear-defending headphones (MR Confon, http://www.mr-confon.de/en/) to 3 different sentences spoken consecutively by the male speaker, masked by the female speaker on half the trials, or 3 consecutive identical pure tones. The first sentence or tone commenced 0.5 s after the onset of the visual cue, and there were 0.5 s separating each of the 3 consecutive stimuli delivered during each trial. The presentation of the stimuli, with intervening periods, was complete within 8 s, after which the scanner was triggered to acquire data. Each participant underwent 2 runs of functional imaging data acquisition, a run consisting of each of the 4 conditions presented 6 times. This required the presentation of 144 sentences with either the male speaking alone with sentence endings that were either predictable (M_ALONE/PRED_) or unpredictable (M_ALONE/NON-PRED_), or in the presence of the female speaker (MF_DIOTIC/PRED_ and MF_DIOTIC/NON-PRED_). As the database only contained 128 sentences, 16 sentences were presented twice. The order of conditions during each run was pseudo-randomized within subjects. The 2 runs were separated by the acquisition of a high-resolution T1-weighted anatomical MR scan.

Following the scanning session, the participants were presented with a forced-choice sentence recognition task on a list of 120 written sentences. Eighty sentences were those spoken by the male speaker during the scanning session, half when he spoke alone and half when his voice was partially masked by the female speaker. An equal number were chosen from those with and without a semantically predictable ending. None of the sentences were drawn from the 16 that had been presented twice. Of the remaining 40 sentences, 20 were those spoken by the female speaker and 20 had not been presented during the scanning session. The participants were required to indicate which sentences they recognized as having been spoken by the male speaker during the scanning session. Subjects were familiarized with the experiment, both with the prompts and with examples of the stimuli. The example stimuli were not used during the scanning session.

#### Image Acquisition

MRI data were obtained on a Philips Intera 3.0 Tesla scanner using dual gradients, a phased-array head coil, and sensitivity encoding with an undersampling factor of 2. Functional magnetic resonance images were obtained using a T2*-weighted gradient-echo, echoplanar imaging (EPI) sequence (repetition time 8 s; acquisition time 2 s; echo time, 30 ms; flip angle, 90°). Thirty-two axial slices with a slice thickness of 3.25 mm and an interslice gap of 0.75 mm were acquired in ascending order (resolution, 2.19 × 2.19 × 4 mm; field of view, 280 × 224 × 128 mm). As described earlier, “sparse” sampling was used so that the subjects heard all stimuli without background scanner noise. To correct for magnetic field inhomogeneities, a quadratic shim gradient was used. In addition, high-resolution (1 mm^3^) T1-weighted structural images were acquired for each subject. The trials were programed using E-prime software (Psychology Software Tools) and then presented on an IFIS-SA system (In Vivo Corporation).

### Experiment 2

#### Stimuli

Five speech auditory conditions were used (Fig. 1). The first auditory condition was a female speaker alone (F_ALONE_). The second was the female speaker in the presence of background babble (F_BABBLE_), with the voice and babble mixed into the same channel to remove spatial cues (diotic presentation). The third was the female speaker in the presence of a male speaker, again with diotic presentation (FM_DIOTIC_). The fourth and fifth conditions had competing female and male speakers, as in the third condition, but in these a simulated azimuth spatial cue was added (dichotic presentation). This was either with the female speaker at 30° to the left and the male speaker at 30° to the right (F_LEFT_M_RIGHT_) of the midline, or vice versa (M_LEFT_F_RIGHT_). Each participant included in this study was rehearsed to ensure that they perceived the intended directionality of the fourth and fifth auditory conditions.

Factual statements, taken from children encyclopedias and books, were spoken by a native English female and male speaker and recorded in an anechoic chamber. The stimuli, edited in Sound Studio 2.2.4 (Felt Tip software www.felttip.com), were 6- to 7-s duration. Babble was created using online audio from the BBC sound effect library (“cocktail party —close perspective and atmosphere”) and cut to the desired length. Spatial cueing for F_LEFT_M_RIGHT_ and M_LEFT_F_RIGHT_ was introduced by manipulating intensity using a public-domain database of high spatial-resolution, head-related transfer functions (CIPIC HRTF database) (Algazi et al. 2001). This simulates the effects of sound scattering due to different pinna, head, and torso dimensions. Stimuli included 384 female and 288 male statements and 48 babble speech, randomly chosen. The stimuli were adjusted using Praat (http://www.fon.hum.uva.nl/praat/) to have the same root-mean-squared average intensity. The female target sentences and matched-length male and babble competing sentences were mixed together at 3:0 dB signal-to-noise ratios, to make hearing the female slightly easier. The participants never heard the same sentence twice at any point during the practice sessions or the tasks.

The instruction to the subjects was the same for all auditory conditions: listen to the female speaker, understand the statement she makes, and prepare to answer a written question (presented in Helvetica, font size 70, on a computer screen, which was projected to a 45° angled mirror 10 cm from the participants eyes), with a “yes” or “no” button press response on the next trial. The response trial (Response) was the sixth condition. For the conditions when there was only a female speaker or a female speaking against background babble, all the questions related to what the female speaker had said accurate responses being equally divided between “yes” and “no.” In the 3 conditions when there was a competing male speaker, half the questions related to what the female speaker had said and half to what the male speaker had said. During each trial with a distracting male speaker, the phrases spoken by the female and male speakers were unrelated in meaning. As an example, the participant heard the female speaker say “She rummaged about in the closet looking for a recipe, turning over all of her mother's recipe books,” while the male speaker said “The white-tailed deer is tan or reddish-brown in the summer and grayish-brown in the winter.” The question in the immediately ensuing trial related either to what the female had said (“She was looking for a recipe?”), or what the male speaker had said (“The deer is white in winter?”). The subjects were not informed beforehand that questions might relate to the content of the speech of the unattended male speaker, to ensure they did not attempt to divide attention between the 2 speakers. Each participant undertook 2 short practice runs of the auditory attention task prior to scanning. The seventh condition was a Silence condition, the same as in the first study.

#### Study Design

The conventional “sparse” sampling used for image acquisition in the first study was modified to further improve signal-to-noise. Interleaved silent steady state (ISSS) imaging was used. This ensured that all stimuli were heard with minimal background scanner noise, while providing greater time course information than conventional sparse scanning (Schwarzbauer et al. 2006). During the ISSS runs, volume acquisition was accomplished using 5 “imaging” volumes followed by 4 “quiet” volumes, giving 10 s of gradient activity followed by 8 s of reduced scanner noise. Radiofrequency (RF) activity, which does not contribute to scanner noise, in the form of adiabatic fat saturation and slice excitation, was continued in all volumes to keep the recovery of the longitudinal magnetization equal throughout all volumes. There was no data acquisition during the quiet volumes as all gradient activity was turned off, aside from the concomitant slice select gradient. The slice select gradient's refocusing lobe was also turned off. The slice select gradient was necessary to keep the selective RF excitation equivalent. This gradient lobe used a 20 mT/m/ms slew rate in all volumes, whereas the peak slew rate in the imaging volumes was 230 mT/m/ms.

For an individual trial, the auditory stimuli were presented during a period of 8 s when there was no data acquisition and much reduced scanner noise. As in the first study, the stimuli were played through ear-defending headphones. Data were then acquired during the ensuing 10 s of the response trial, consisting of 5 TRs, each of 2 s duration. Once the auditory stimulus was delivered, a jitter period (averaging 2 s across the trials) occurred before the visual question would appear and remain for 7–9 s during the response trial, allowing the participants’ time to read the question and respond with a “yes/no” button press. Each condition, including Silence, was presented as a block of 4 consecutive trials, presented twice during each run. There were 2 runs, with the order of conditions during each run pseudo-randomized within subjects.

#### Image Acquisition

Access to the scanner used for the first experiment was no longer feasible at the time of the second study, and so the second study was performed on an alternative 3T scanner. T2*-weighted gradient-echo planar images were collected on a 3T Siemens Tim Trio scanner with a 12-channel phased-array head coil. Thirty-five contiguous axial slices at each of 2 echo times (13 and 31 ms) with a slice thickness of 3 mm were acquired in interleaved order (resolution, 3 × 3 × 3 mm; field of view, 192 × 192 × 105 mm), with a repetition time of 2 s, and 242 volumes were acquired in 14 m:42 s. To correct for magnetic field inhomogeneities, the manufacturer-provided higher-order shim procedure was used. High-resolution (1 mm^3^) T1-weighted structural images were also acquired for each subject. Stimuli were presented using the Psychophysics Toolbox (Brainard 1997) under MATLAB (Mathworks).

### Data Analysis

#### Univariate Whole-Brain Analyses

For both studies, these analyses were carried out within the framework of the general linear model using FEAT (FMRI Expert Analysis Tool) Version 5.98, part of FSL (FMRIB's Software Library, http://www.fmrib.ox.ac.uk/fsl). The following image preprocessing steps were applied: realignment of EPI images for motion correction using MCFLIRT (Jenkinson et al. 2002); nonbrain removal using BET (Brain Extraction Tool) (Smith 2002); spatial smoothing using a 6-mm full-width half-maximum Gaussian kernel; grand-mean intensity normalization of the entire four-dimensional dataset by a single multiplicative factor; and high-pass temporal filtering (Gaussian-weighted least-squares straight line fitting, with sigma = 50 s) to correct for baseline drifts in the signal. Time series statistical analysis was carried out using FILM (FMRIB's Improved Linear Modeling) with local autocorrelation correction. Registration to high-resolution structural and Montreal Neurological Institute (MNI) standard space images (MNI 152) were carried out using FMRIB's Linear Image Registration Tool (FLIRT). *Z* (Gaussianized *T/F*) statistic images were threshold using clusters determined by *Z* > 2.3 and a corrected cluster significance threshold of *P* = 0.05.

The combination of the different runs at the individual subject level was carried out using a fixed-effects model. Individual design matrices were created, modeling the different behavioral conditions. Contrast images of interest in each study were produced from these individual analyses and used in the second-level higher analysis. Higher-level between-subject analysis was carried out using a mixed-effects analysis with the FLAME (FMRIB's Local Analysis of Mixed Effects) tool, part of FSL. Final statistical images were corrected for multiple comparisons using Gaussian Random Field-based cluster inference with a height threshold of *Z* > 2.3 and a cluster significance threshold of *P* < 0.05.

In the first study, 1 TR was acquired at the end of each trial, and the recorded signal will have been an accurate representation of the net neural activity in response to whichever stimulus had been delivered over the preceding 8 s. The second study required a more complex analysis, as 5 TRs were acquired during the response trials. To ensure accurate allocation of the TRs to specific stimulus- or response-evoked hemodynamic response functions (HRFs), individual time series explanatory variables (EVs) were generated using the tools from the FSL library (glm_gui). Three column format data were entered to produce a single-column time series EV that was used in the remaining analysis. For the auditory conditions, the columns included timing for when the sound started and its duration, whereas for the response period, it included the onset of the question and the duration it remained on the screen. This allowed a design that accurately represented the timing of the scanning protocol, to ensure the analysis weighted the HRFs evoked by listening and responding toward their appropriate conditions. Thus, the design matrix modeled the first TR strongly toward listening; the fifth TR strongly toward reading the question, deciding the answer and responding based on what had been heard in the previous trial and held in working memory; with the other 3 TRs weighted appropriately in between these 2 extremes.

The data were analyzed using a standard random-effects general linear model, using tools from the FSL library (FEAT version 5.98) (Smith et al. 2004). After image preprocessing, which required anatomical normalization with realignment of the EPI images, removing motion effects between scans and smoothing to 5-mm full-width half-maximum Gaussian kernel, the data were entered into a univariate statistical analysis within FSL, based on the general linear model. Within the design matrix, the 4 auditory verbal conditions were entered into a factorial analysis of variance. Main effects and interactions were thresholded (*Z* > 2.3) with a cluster significance threshold of *P* < 0.05 to correct for whole-brain analyses (Beckmann et al. 2003).

#### Independent Component Analysis

For each study, this was carried out using group temporal concatenation probabilistic independent component analysis (ICA) implemented in MELODIC (Multivariate Exploratory Linear Decomposition into Independent Components) Version 3.10, part of the FSL software (Beckmann and Smith 2004). This approach to the ICA was used rather than tensor-ICA (Beckmann and Smith 2005), as the temporal presentation of the stimuli was different between subjects. Such multivariate analysis can extract important information from the data that are not always apparent from a subtractive univariate analysis (for example, Leech et al. 2012). ICA takes advantage of low-frequency fluctuations in the fMRI data to separate the signal into spatially distinct components. A particular advantage of ICA, which increases sensitivity when detecting net regional neural responses, is controlling for time series unrelated to brain function. These will be identified as separate components; for example, movement-related artifact not removed by the initial image preprocessing.

Data preprocessing for the ICA included masking of nonbrain voxels, voxel-wise de-meaning of the data, and normalization of the voxel-wise variance of the noise. The ICA for each study was set up to decompose the data into 20 independent components containing distributed neural networks, movement artifact, and physiological noise. The choice of the number of component maps reflects a tradeoff between granularity and noise. It is motivated by the attempt to maximize the homogeneity of function within each network while maximizing the heterogeneity between them. Previous applications of ICA to fMRI data have used 20–30 component maps (Beckmann et al. 2005; Smith et al. 2009; Leech et al. 2011), and this study adopted the same approach.

The data were projected into a 20-dimensional subspace using principal component analysis. The whitened observations were decomposed into a set of 20 component maps and associated vectors describing the temporal variations across all runs and subjects by optimizing for non-Gaussian spatial source distributions using a fixed-point iteration technique (Hyvärinen 1999). Estimated component maps were divided by the standard deviation of the residual noise and thresholded by fitting a Gaussian/Gamma mixture model to the histogram of intensity values (Beckmann and Smith 2004).

#### Region-of-Interest Analysis

This post hoc analysis was performed to relate activity in a ventral right frontoparietal network across all the auditory conditions in the second study and relate activity generated in these regions to those in the first study. A right frontal and right inferior parietal ROI were defined from activated regions evident in the univariate contrasts from the first study and applied to the second (see later). The mean effect size in each of the listening conditions, relative to rest, was determined for each functionally defined ROI. These means were plotted as bar charts with 95% confidence intervals.

Ideally, a direct whole-brain comparison between the 2 studies would be the preferred method to ROI analyses. There are issues concerning different signal-to-noise characteristics between scanners, which to resolve completely would require complex analyses of individual scanner performances. Nevertheless, it has been proposed that between-group analyses, when the data for the groups have been acquired on different scanners, adds relatively little to the variance in the BOLD signal (Bennett and Miller 2010). Therefore, we performed a whole-brain comparison in addition to the ROI analyses. When performing this direct comparison between groups, we entered the scanner as a covariate in the design matrix.

## Results

### Study 1

#### Behavioral

A d’ signal-detection measure (controlling for response bias) was used in the analyses of the forced-choice recognition memory test at the end of the scanning session. Subjects performed better than chance for all sentence types spoken by the male (two-tailed Student's *t*-tests, for all 4 stimulus types: Female vs. M_ALONE/PRED_ *t*_28_ = −14.4, *P* < 0.0001; Female vs. M_ALONE/NON-PRED_ *t*_28_ = −8.8, *P* < 0.0001; Female vs. MF_DIOTIC/PRED_ *t*_28_ = −11.5, *P* < 0.0001; Female vs. MF_DIOTIC/NON-PRED_ *t*_28_ = −8.3, *P* < 0.0001). The mean results for correctly identifying the male sentences were as follows: (M_ALONE/PRED_) = 74.5%, (M_ALONE/NON-PRED_) = 55.5%, (MF_DIOTIC/PRED_) = 55.7%, and (MF_DIOTIC/NON-PRED_) = 46.9%. A 2 × 2 repeated-measures analysis-of-variance (ANOVA), with the factors diotic/single speech and semantically predictable/unpredictable sentence ending, was performed. When the male spoke alone, the sentences were remembered significantly better than when there was distraction by the female speaker (*F*_1,28_ = 4.7, *P* < 0.05), and the sentences with predictable sentence endings were remembered significantly better than those with unpredictable endings (*F*_1,28_ = 18.2, *P* < 0.001). There was no significant interaction between the 2 factors (*F*_1,28_ < 2, *P* > 0.1).

#### Univariate Whole-Brain Analysis

**Figure 2.**
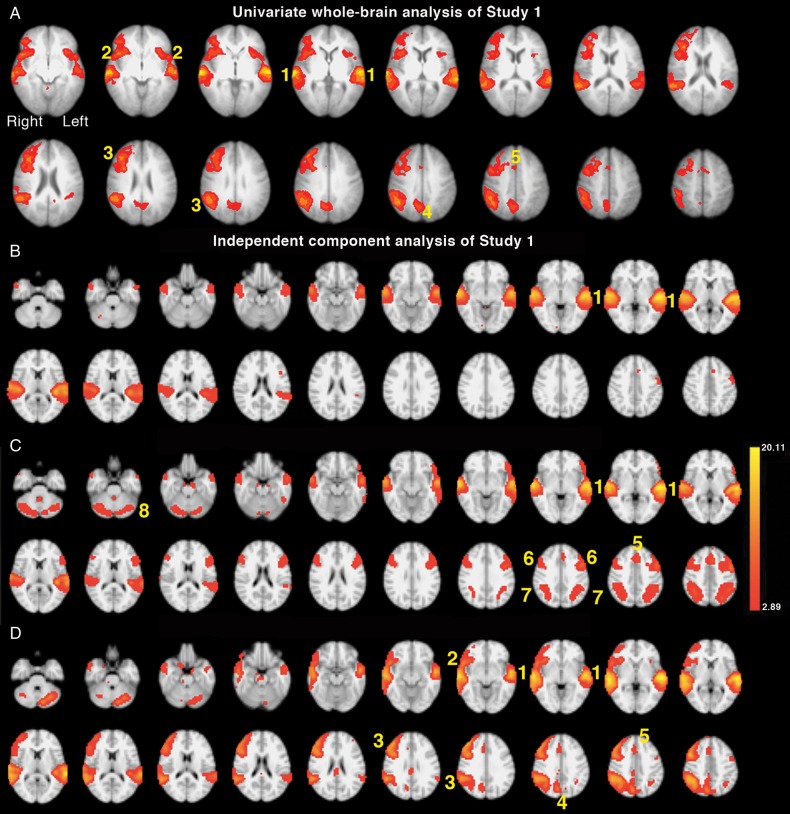
Axial slices are shown in radiological convention, that is the right hemisphere on the left of each slice, beginning with the most ventral slice. (*A*) Univariate whole-brain analysis of Study 1. The significant main effect of competing speech (MF_DIOTIC/NON-PREDICTABLE_ + MF_DIOTIC/PREDICATBLE_) contrasted with noncompeting (M_ALONE/NON-PREDICTABLE_ + M_ALONE/PREDICTABLE_) speech is projected as a red/yellow overlay, with a voxel-level threshold *Z* > 2.3, cluster-level threshold, *P* < 0.05. (1) Superior temporal gyri (STG); (2) anterior insula and frontal operculum (aI/FOp); (3) lateral prefrontal and inferior parietal cortical network (MFG/SMG); (4) precuneus; (5) dorsal anterior cingulate cortex and adjacent superior frontal gyrus (dACC/SFG). (*B–D*) Results from the 20-component independent-component analysis (ICA). (*B*). Component 2 demonstrated regions with significant activity during all listening conditions (including Tones) > Silence (*P* < 0.00001). (1) Bilateral STG. (*C*). Component 3 demonstrated areas of significant activity for all speech listening conditions > Silence (*P* < 0.00001). (1) Bilateral STG, (5) dACC/SFG, (6) Bilateral inferior frontal sulci (IFS), (7) bilateral intraparietal sulcus (IPS), (8) lateral cerebellar hemispheres. (*D*). Component 4 demonstrated a main effect of diotic speech (MF_DIOTIC/NON-PREDICTABLE_ + MF_DIOTIC/PREDICATBLE_) > single speech (M_ALONE/NON-PREDICATBLE_ + M_ALONE/PREDICTABLE_) (*P* < 0.00007). (1) Bilateral STG, (2) right aI/FOp, (3) MFG/SMG, (4) precuneus, (5) dACC/SFG.

The data were entered into a 2 (MF_DIOTIC/PRED_ and MF_DIOTIC/NON-PRED_) × 2 (M_ALONE/PRED_ and M_ALONE/NON-PRED_) ANOVA. There was a significant main effect of listening to diotic compared with single speech (Fig. 2*A*). The regions with significantly greater activity were as follows: bilateral superior temporal gyri (STG), the dorsal anterior cingulate cortex and adjacent medial aspect of the superior frontal gyrus (dACC/SFG), and bilateral anterior insular cortices and adjacent frontal opercula (aI/FOp), the so-called cingulo-opercular network; an extensive right lateral prefrontal and inferior parietal cortical network, centered on the posterior middle frontal and supramarginal gyri (MFG/SMG), respectively; and a posterior midline region, within the precuneus. There was no main effect of the semantic predictability of sentence ending, and there were no significant interactions.

#### Multivariate Whole-Brain Analysis

The data were entered into an independent components analysis (ICA), specifying 20 components. Nine contrasts between conditions were chosen a priori (Tone > Rest; All speech > Rest; [M_ALONE/PRED_ + M_ALONE/NON-PRED_] > Rest; [M_ALONE/PRED_ + M_ALONE/NON-PRED_] > Tone; [MF_DIOTIC/PRED_ + MF_DIOTIC/NON-PRED_] > Tone; [MF_DIOTIC/PRED_ + MF_DIOTIC/NON-PRED_] > Rest; [M_ALONE/NON-PRED_ + MF_DIOTIC/NON-PRED_] > [M_ALONE/PRED_ + MF_DIOTIC/PRED_]; [M_ALONE/PRED_ + MF_DIOTIC/PRED_] > [M_ALONE/NON-PRED_ + MF_DIOTIC/NON-PRED_]; [MF_DIOTIC/PRED_ + MF_DIOTIC/NON-PRED_] > [M_ALONE/PRED_ + M_ALONE/NON-PRED_]), and significance was set at *P* < 0.005 Bonferroni-correcting for multiple contrasts. Components where most or all of the signal was confined to edges of the brain, or was located within the ventricular systems and white matter, were discarded as related to motion or other artifacts. From the remaining components, the 3 that demonstrated significant differences between conditions are presented here (Fig. 2*B–D*) (see Supplementary, Table 1, for “centre of mass” coordinates).

Component 2 (Fig. 2*B*) demonstrated a hierarchy of activation between conditions, all significant at *P* < 0.00001: [Tones > Silence], [(M_ALONE/PRED_ + M_ALONE/NON-PRED_) > Tones], and [(MF_DIOTIC/PRED_ + MF_DIOTIC/NON-PRED_) > (M_ALONE/PRED_ + M_ALONE/NON-PRED_)]. The majority of the activity was distributed along the left and right STG (primary and association auditory cortices).

Component 3 (Fig. 2*C*) also contained activity along the left and right STG. However, activity that correlated with this subsystem within auditory association cortex was observed in the dACC/SFG, the left inferior frontal gyrus (IFG), and between the left and right inferior frontal and intraparietal sulci (IFS/IPS). There was additional activity in both lateral cerebellar hemispheres, and the dACC/SFG and IFS/IPS networks have been described as having common functional connections with the cerebellum (Dosenbach et al. 2008). There was 1 lateralized region, in the left posterior middle and inferior temporal gyri. For Component 3, activity during the 2 main speech conditions was greater than Silence, significant at *P* < 0.000001: [(M_ALONE/PRED_ + M_ALONE/NON-PRED_) > Silence] and [(MF_DIOTIC/PRED_ + MF_DIOTIC/NON-PRED_) > Silence]. Activity was also greater for the contrast of listening to Tones with Silence, *P* = 0.009. Other contrasts were not significant, correcting for multiple contrasts.

Component 4 (Fig. 2*D*) comprised activity that had a distribution similar to that observed as the main effect of listening to 2 speakers compared with single speech in the univariate analysis. Activity in this component was significantly different between listening when there were 2 speakers compared with 1: [(MF_DIOTIC/PRED_ + MF_DIOTIC/NON-PRED_) > (M_ALONE/PRED_ + M_ALONE/NON-PRED_)], *P* = 0.00007.

No component demonstrated any effect of the semantic predictability of sentence endings [(M_ALONE/NON-PRED_ + MF_DIOTIC/NON-PRED_) > or < (M_ALONE/PRED_ + MF_DIOTIC/PRED_)]. The inclusion of this experimental manipulation into the design had been to observe whether a reaction to an unanticipated stimulus ending modulated the response of higher-order cortices involved with the cognitive control and attention involved in listening to speech. In the absence of any observable modulation, this experimental manipulation is not considered further.

#### Summary of Findings From Study 1

Component 2 of the ICA analysis demonstrated that bilateral primary and association auditory cortex responded in a “bottom-up” manner to stimuli of increasing auditory complexity: Silence << Tones << M_ALONE_ << MF_DIOTIC_. Components 3 and 4 demonstrated that overlapping networks within auditory cortex were also demonstrating correlated activity with multiple higher-order systems: the bilateral cingulo-opercular and IFS/IPS networks, and the ventral right frontoparietal network (MFG/SMG) and precuneus that was also evident as the main effect of listening to 2 speakers in the univariate whole-brain ANOVA. Therefore, activity within auditory cortex was simultaneously influenced by both the complexity of ascending auditory signal and by top-down signal from networks that have been associated with attention and cognitive control. However, there was a dissociation of activity across these high-order networks, most evident in their visualization as separate components within the ICA. The cingulo-opercular and IFS/IPS networks activated together and responded to any listening condition, including when the participants heard pure tones without an explicit task demand. Any difference in activity within these networks between listening to MF_DIOTIC_ and to M_ALONE_ was small, but much more evident in the network consisting of right MFG/SMG and the precuneus. There was anatomical overlap between these 2 broad networks, in both STG, right IFS/IPS and in dACC/SFG.

### Study 2

#### Behavioral

A pilot study on 5 subjects was performed to determine whether the probe questions could be answered above chance through using prior knowledge, even though the questions had been designed to relate specifically to the previous statement by the female or male speaker. For all female and all male sentences, the mean responses (39–49%) were not above chance (50%). In contrast, the participants during scanning in response to questions on statements spoken by the female were all significantly more accurate than chance (*P* < 0.0001). However, a repeated-measures ANOVA showed a significant difference in accuracy between conditions (*F*_1,24_ = 8.604, *P* < 0.0001). One-sample *t*-tests, Bonferroni corrected for multiple comparisons, demonstrated that F_SINGLE_ = F_BABBLE_ = M_LEFT_F_RIGHT_ (*P* > 0.5). However, F_ALONE_ > FM_DIOTIC_ (*P* < 0.05), >F_LEFT_M_RIGHT_ (*P* < 0.0001), and M_LEFT_F_RIGHT_ > F_LEFT_M_RIGHT_ (*P* < 0.05). Therefore, accuracy on questions about the female statements was not statistically different across both masked and unmasked conditions except for a small but significant decline on FM_DIOTIC_ and a greater decline on F_LEFT_M_RIGHT_. In the latter condition, the “attended” speech was directed toward the right hemisphere, nondominant for language.

The responses to questions on statements spoken by the “unattended” male speaker were significantly more accurate than chance during FM_DIOTIC_ and F_LEFT_M_RIGHT_ (*P* < 0.0001), although a little below chance during M_LEFT_F_RIGHT_ (mean 43%, chance 50%, *t* = 2.25*, P* < 0.05). A repeated-measures ANOVA on the response to the sentences spoken by the male speaker during FM_DIOTIC_, M_LEFT_F_RIGHT_, and F_LEFT_M_RIGHT_ demonstrated a significant difference in accuracy between conditions (*F*_1,24_ = 30.5, *P* < 0.0001). One-sample *t*-tests, Bonferroni corrected for multiple comparisons, demonstrated that FM_DIOTIC_ = F_LEFT_M_RIGHT_ (*P* > 0.5), but F_LEFT_M_RIGHT_ > M_LEFT_F_RIGHT_ (*P* < 0.0001).

In summary, the subjects did attend to the female speaker in all conditions but found it most difficult when the female was presented to the left ear, and therefore, predominantly to the right cerebral hemisphere. There are limitations to introducing spatial cues using simulated head-related transfer functions (HRTFs) (Algazi et al. 2001), which will have deviated to a variable extent from the HRTF of individual subjects, resulting in weaker dichotic/diotic contrasts than could be obtained with listening conditions in free field or with individually determined HRTFs. Nevertheless, there was a significant behavioral effect, with more correct responses when the female speaker was “located” to the right rather than the left of the participants. Further, responses to what the “unattended” male speaker had said were least when his voice was presented to the left (that is, predominantly to the right hemisphere) compared with both the FM_DIOTIC_ and M_LEFT_F_RIGHT_ conditions. Therefore, spatial cues were perceived by the participants during the dichotic listening conditions.

#### Univariate Whole-Brain Analysis

**Figure 3.**
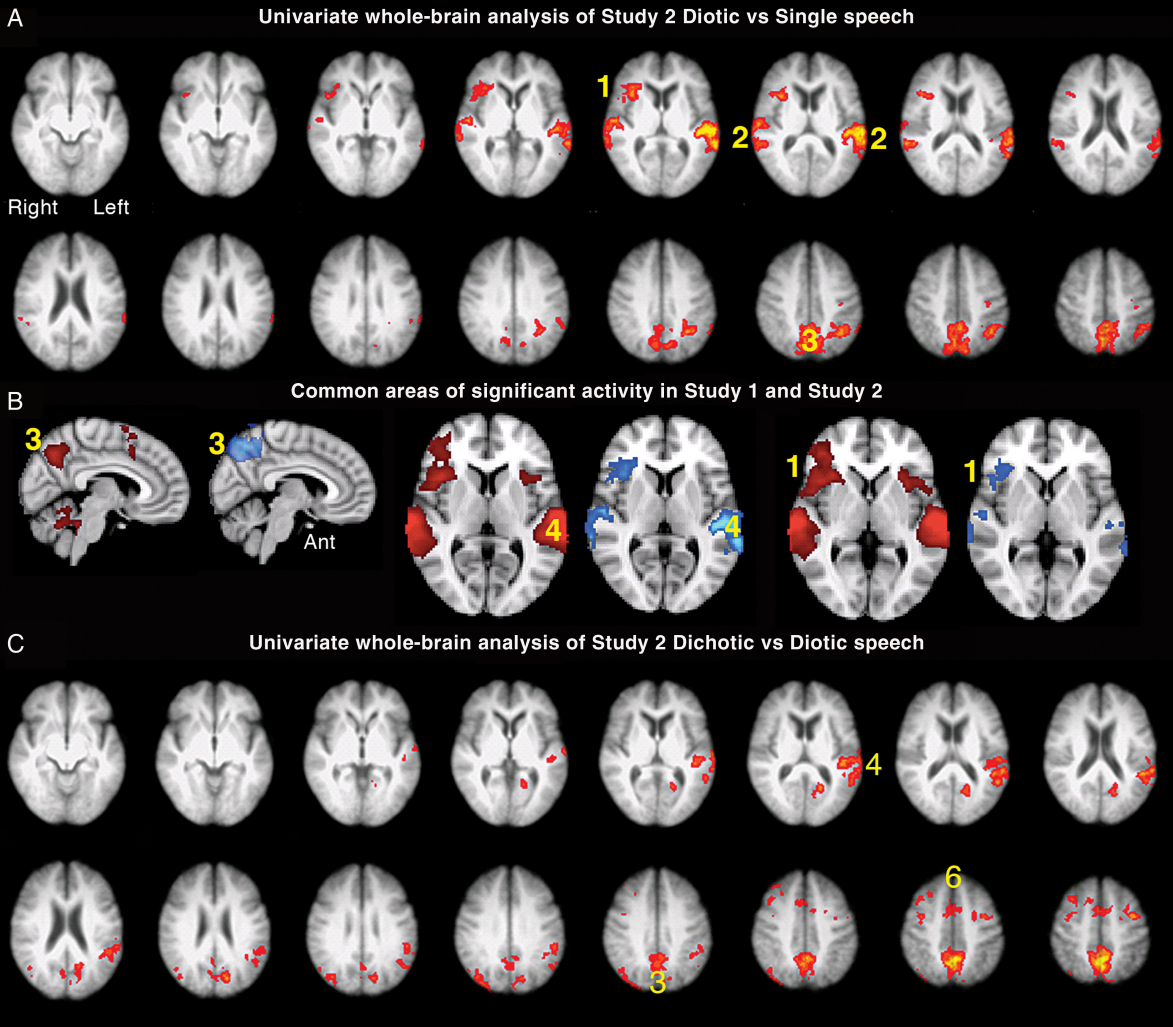
Axial slices displayed as in Figure 2. (*A*). Univariate whole-brain analysis of Study 2. There was a significant main effect of diotic (F_BABBLE_ + FM_DIOTIC_) contrasted with single speech (F_ALONE_) speech projected as a red/yellow overlay, with a voxel-level threshold *Z* > 2.3, cluster-level threshold *P* < 0.05. (1) Anterior insular/inferior frontal cortex (aI/IFG), (2) left planum temporale and adjacent anterior inferior parietal lobe (PT/IPL) and right posterior superior temporal gyrus (STG), (3) precuneus, (*B*) sagittal and axial views showing regions of common significant activity generated from the univariate analysis contrasting diotic speech to single speech, projected as red/yellow overlay in Study 1 and blue overlay in Study 2, with a voxel-level threshold *Z* > 2.3, cluster-level threshold *P* < 0.05. (1) Right aI/IFG, (3) (displayed on midline sagittal views) precuneus, (4) Left PT/IPL. (*C*). Demonstrates axial slices from the univariate contrast of Dichotic (M_LEFT_F_RIGHT_ + F_LEFT_M_RIGHT_) > Diotic (FM_DIOTIC_). (3) Precuneus, (4) Left PT/IPL; (6) anterior cingulate cortex (ACC) and probably activity localized to frontal eye and supplementary eye fields.

The first analysis was a contrast of the 2 diotic listening conditions (F_BABBLE_ + FM_DIOTIC_) with F_ALONE_. This demonstrated a reduced distribution of activity compared with the univariate contrast of MF_DIOTIC_ with M_ALONE_ in the first study, with activity confined to the right aI/IFG, left planum temporale and adjacent anterior inferior parietal lobe (PT/IPL), right posterolateral STG, left IPS, and precuneus (Fig. 3*A*). These regions, with the exception of the left IPS, were also evident in the contrast of MF_DIOTIC_ with M_ALONE_ in the first study (Fig. 3*B*). When the 2 conditions with spatial cues (M_LEFT_F_RIGHT_ and F_LEFT_M_RIGHT_) were each contrasted with FM_DIOTIC_, it was evident that these 2 conditions with spatial cues resulted in greater activity in the precuneus, the left PT/IPL and the dACC/SFG (Fig. 3*C*). Therefore, speech-masked-by-speech without spatial cues activated these 2 regions relative to F_ALONE_, but activity increased significantly in the presence of spatial cues. Activity in these regions was not significantly greater if the female speaker was presented to the right or left ear during the dichotic listening conditions. The spatial cues also resulted in greater activity in anterior regions associated with eye movements, the frontal eye fields (Fig. 3*C*), which form part of the so-called dorsal attentional network (Corbetta et al. 2008).

**Figure 4.**
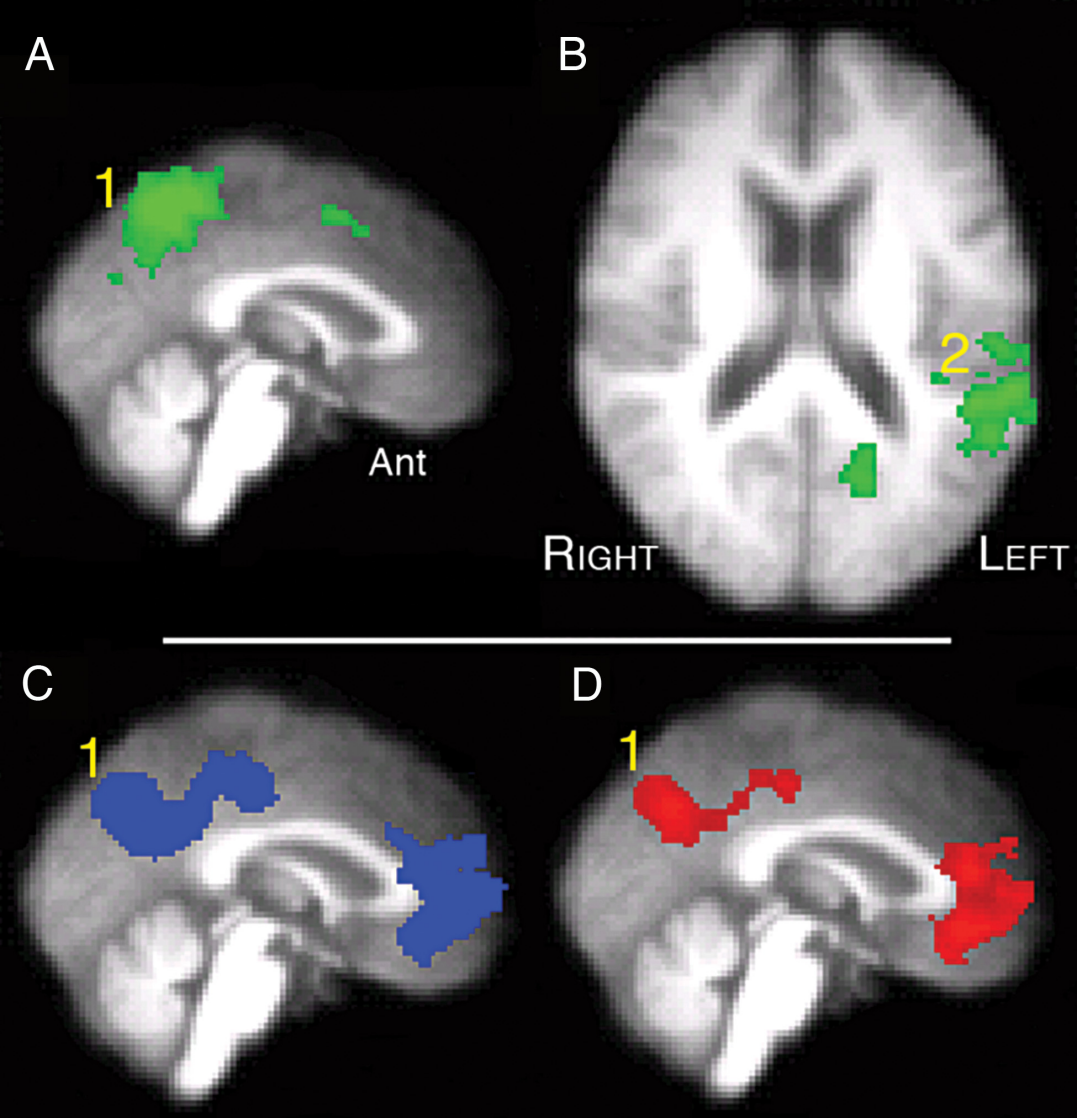
Univariate whole-brain analysis of Study 2, exploring 3 contrasts with a voxel-level threshold *Z* > 2.3 and cluster-level threshold, *P* < 0.05. (*A*) (sagittal midline projection) and (*B*) (axial section): dichotic (F_LEFT_M_RIGHT_ + M_LEFT_F_RIGHT_) against diotic (FM_DIOTIC_ + F_BABBLE_) projected as a green overlay. (1) precuneus, (2) left planum temporale and adjacent anterior inferior parietal lobe (PT/IPL). (*C*) (sagittal midline projection): rest against Diotic projected as a blue overlay. (1) Precuneus and adjacent posterior cingulate cortex (PCC). (*D*) (Sagittal midline projection): Rest against Dichotic projected as a red overlay. (1) Precuneus and PCC.

Networks for attention and cognitive control demonstrate anticorrelated activity with the default mode network (DMN), a system that is most active during “Rest” states and deactivated by attending and responding to external stimuli (Fox et al. 2005). A prominent posterior component of the DMN is located centered on the posterior cingulate cortex. Figure 4 demonstrates the contrast of M_LEFT_F_RIGHT_ + F_LEFT_M_RIGHT_ with the rest condition (Silence) and vice versa. The posterior midline activity associated with attending to one speaker in the presence of another, most evident when spatial cues were included, was located dorsal to the midline posterior component of the DMN.

#### Multivariate Whole-Brain Analysis

An independent components analysis (ICA), specifying 20 components to all trials, was performed. Of the multiple contrasts between conditions, 11 contrasts were chosen a priori (F_ALONE_ > Rest; F_BABBLE_ > F_ALONE_; FM_DIOTIC_ > F_ALONE_; M_LEFT_F_RIGHT_ > F_ALONE_; F_LEFT_M_RIGHT_ > F_ALONE_; FM_DIOTIC_ > F_BABBLE_; [M_LEFT_F_RIGHT_ F_LEFT_M_RIGHT_] > [FM_DIOTIC_ + F_BABBLE_]; [FM_DIOTIC_ + F_BABBLE_] > [M_LEFT_F_RIGHT_ F_LEFT_M_RIGHT_]; F_LEFT_M_RIGHT_ > M_LEFT_F_RIGHT_; M_LEFT_F_RIGHT_ > F_LEFT_M_RIGHT_; [F_ALONE_ + FM_DIOTIC_ + F_BABBLE_ + M_LEFT_F_RIGHT_] > F_LEFT_M_RIGHT_) with a Bonferroni-corrected significance level set at *P* < 0.005 (see Supplementary, Table 1, for “center of mass” coordinates).

**Figure 5.**
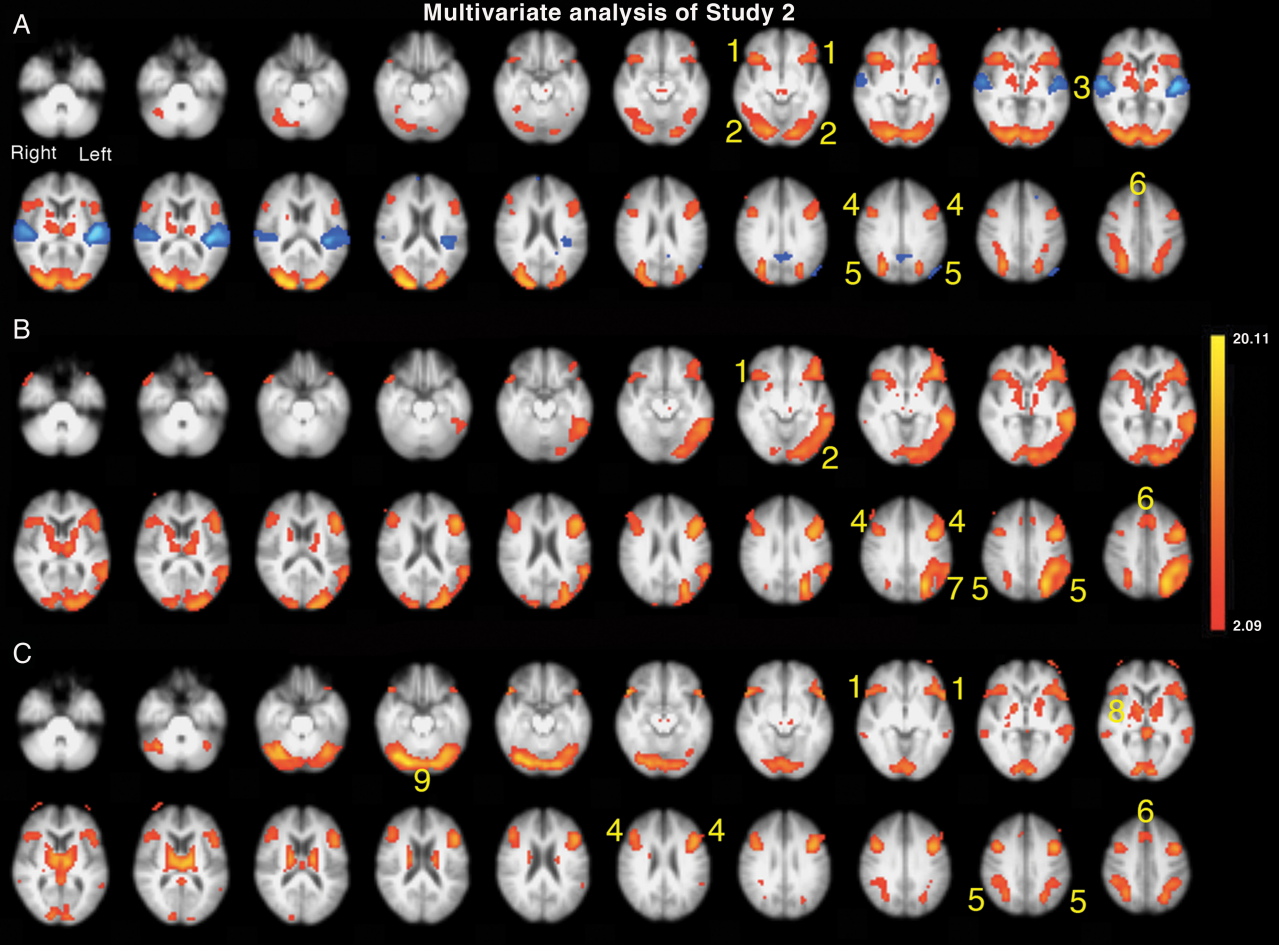
Axial slices displayed as in Figure 2. Multivariate analysis of Study 2, specifying 20 components, with regions of significant activity displayed as red/yellow overlays. (*A*). Component 2 demonstrated activity for Response > Listening in (1) bilateral anterior insulae and frontal opercula aI/FOp, (2) visual cortex (4) bilateral inferior frontal sulci (IFS), (5) bilateral intraparietal sulci (IPS), (6) dorsal anterior cingulate cortex and adjacent superior frontal gyrus (dACC/SFG). This activity was anticorrelated with (3) bilateral STG, projected as a blue overlay. (*B*). Component 3 demonstrated activity for Response > all speech Listening conditions combined, all Listening conditions combined > Silence and F_ALONE_ > Silence. (1) aI/Fop, (2) visual cortex, (4) IFS, (5) IPS, (6) ACC, (7) left inferior parietal cortex. (*C*) Component 4 demonstrated a similar hierarchy of activity across conditions observed in Component 3. (1) aI/FOp, (4) IFS, (5) IPS, (6) ACC, (8) basal ganglia and thalami, (9) lateral cerebellum.

Component 1 (not illustrated) showed the expected activity in bilateral STG, with all the Listening trials combined > Rest (*P* < 0.00001), and the diotic and dichotic listening trials each > F_ALONE_ (*P* < 0.0001), but there was no difference between Response (during which no external or self-generated speech was heard) and Silence (*P* = 1). Components 2–4 contained data relevant to activity within the cingulo-opercular and frontoparietal networks. In Component 2 (Fig. 5*A*), the activity was specific for the Response trials, with Response > all the Listening trials (*P* < 0.00001) and Response > Silence (*P* < 0.00001), but activity for all the Listening trials combined was no greater than Silence (*P* = 1). Activity was distributed between the cingulo-opercular and IFS/IPS networks, and the lateral cerebellar hemispheres. In addition, there was activity in the primary and association visual cortices (as the participants had to respond to written questions). There was anticorrelated activity in both STG, consistent with the absence of auditory input during the Response trials. In Component 3 (Fig. 5*B*), activity during the Response trials was > all the Listening trials combined (*P* < 0.00001), but activity during all the Listening trials combined > Silence (*P* < 0.00001), and F_ALONE_ > Silence (*P* < 0.00001). There was no difference in activity between any of the individual Listening trials, with the exception of F_LEFT_M_RIGHT_ > M_LEFT_F_RIGHT_ (*P* = 0.002), the former condition being the one in which the participants were least successful in attending to the female speaker. Although there was activity in the cingulo-opercular and IFS/IPS networks, as in Component 4 (Fig. 5*C*), the prominent activity in both cerebellar hemispheres was absent, and there was strongly left-lateralized activity in the left IFG, posterior inferolateral temporal lobe, and inferior parietal cortex. The only prominent right cortical activity was centered on the posterior MFG. In Component 4 (Fig. 5*C*), the hierarchy of activity was very similar to that observed in Component 3 (Response trials > all the Listening trials combined [*P* < 0.00001]; Listening trials combined > Silence [*P* < 0.00001]; and F_ALONE_ > Silence [*P* < 0.00001]). Again there was no difference in activity between any of the individual Listening trials (*P* > 0.15). The distribution of activity in Component 4 (Fig. 5*C*) was closely similar to that in Component 2, but with little activity in visual cortex and no anticorrelated activity in the STG, but greater activity evident in the basal ganglia and thalami. There was no component demonstrating activity in right inferior parietal cortex, in marked contrast to the results from the first study.

#### ROI Analysis

**Figure 6.**
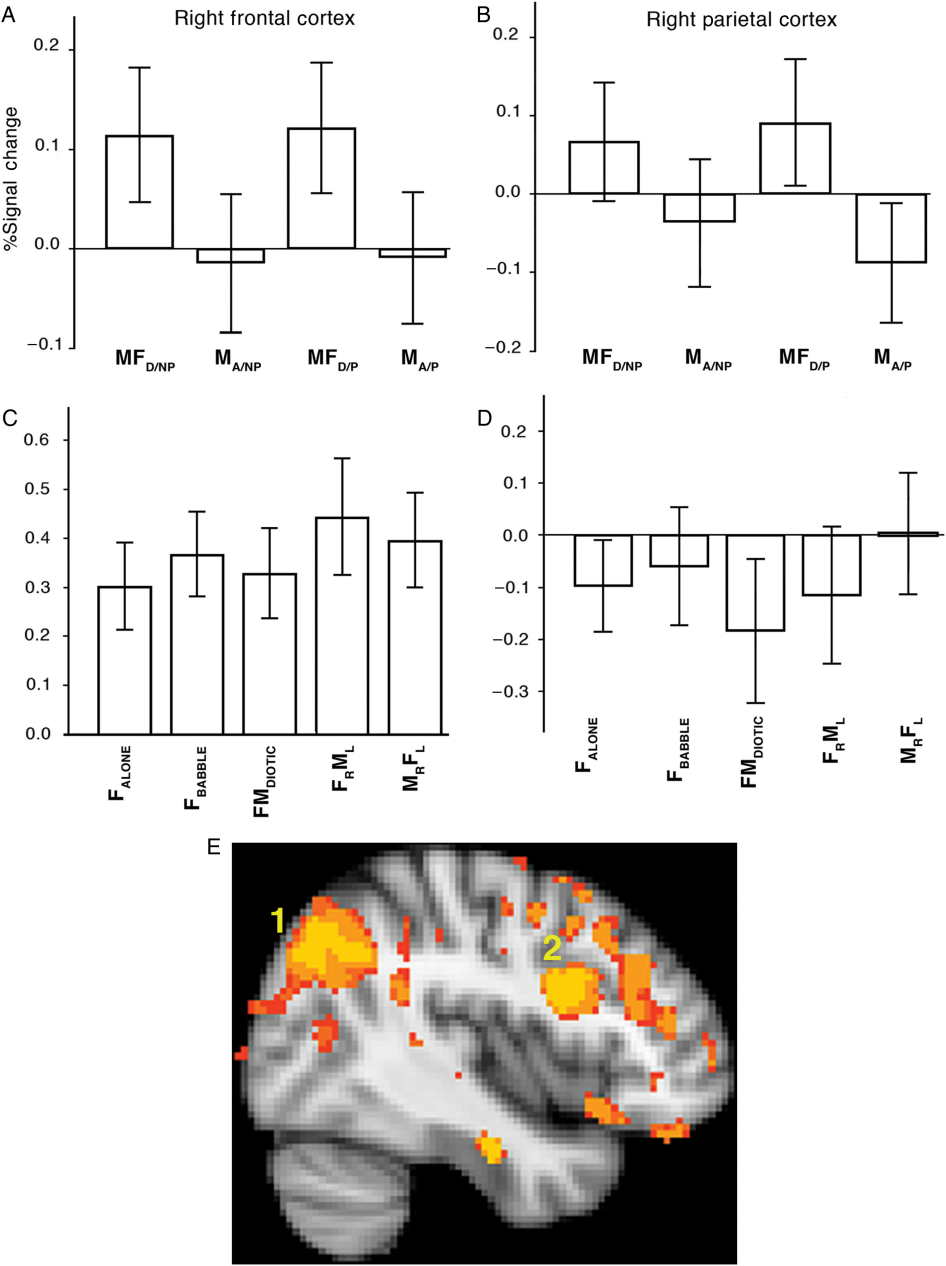
Region-of-interest analysis: (*A–D*) Percentage blood oxygen-level-dependent signal changes for each condition relative to the rest baseline condition. Error bars are the 95% confidence intervals. Study 1 is depicted in Panels (*A*) and (*B*); Study 2 is depicted in Panels *C* and *D*. (*A*) and (*C*) The results from the ROIs in the right middle frontal gyrus (MFG) and (*B*) and (*D*) from the ROIs in the right supramarginal gyrus (SMG). Conditions labeled as in the text, but with the following abbreviations: MF_D/NP_ = (MF_DIOTIC/NON-PREDICTABLE_); M_A/NP_ = (M_ALONE/NON-PREDICTABLE_); MF_D/P_ = (MF_DIOTIC/PREDICTABLE_); MF_A/P_ = (MF_ALONE/PREDICTABLE_), L = left; R = right. (*E*). A sagittal view of the right hemisphere, at *X* coordinate = 42 mm. The contrast of diotic with single speaking conditions was directly compared in whole-brain analyses between Studies 1 and 2. Predictably, from the profile of activities observed in the ROI analyses, both right frontal (labeled 2) and parietal cortices (labeled 1) were more “active” in Study 1. However, this disguises the dissociation of responses between frontal and parietal cortices: The loss of “contrast” in Study 2 was due to an increase in activity in response to a single speaker in frontal cortex_,_ but a decline in activity in response to diotic listening in parietal cortex.

An initial comparison between studies employed region-of-interest (ROI) data from the listening trials. The peaks of activity in the posterior right MFG and the right SMG from the first study were defined, and 8-mm spheres as ROIs were used to extract the signal across conditions from both the first and second studies. The results are presented as bar plots in Figure 6, with 95% confidence intervals. These plots illustrate a clear dissociation of activity across the 2 studies in both ROIs. Within the right frontal region, the dissociation between the 2 studies was due to the different response to the single speaker as a result of the change in task demand: There was increased activity relative to rest, and on a par with that elicited by the diotic and dichotic listening tasks, in the second study. In contrast, in the right SMG, there was no response to any of the auditory conditions during the second study relative to Silence, a major change from the response of this region to speech-masked-by-speech in the first study. Although using the peaks of activity from the first study to interrogate data from the second biases this comparison, and so no direct statistical analyses were applied to the data presented in Figure 6, it was apparent that the right frontal and right parietal regions retuned different profiles of activity across the tasks in the 2 studies. Thus, the loss of differential activity in right frontal cortex in the second study was due to increased activity during F_ALONE_ as the consequence of a task that loaded working memory. In contrast, this task demand resulted in reduced activity across all listening tasks in right inferior parietal cortex.

A whole-brain comparison between the 2 studies was performed, correcting for multiple comparisons, in which MF_DIOTIC_ versus M_ALONE_ in the first study was compared with FM_DIOTIC_ versus F_ALONE_ in the second study, entering “scanner” (+1, −1) as a covariate in the design matrix. This demonstrated the greater right frontoparietal activity in the first study compared with the second, correcting for multiple comparisons, as shown in Figure 6*E*. However, and predictably from the profiles of activity from the ROI data, this contrast failed to demonstrate the dissociation of responses between listening conditions in right frontal and right inferior parietal cortices in the second study as the result of loading working memory. Therefore, the ROI data were essential to interrogate in detail the outcome of the whole-brain comparisons between studies.

#### Contrasting Correct and Incorrect Responses for Study 2

**Figure 7.**
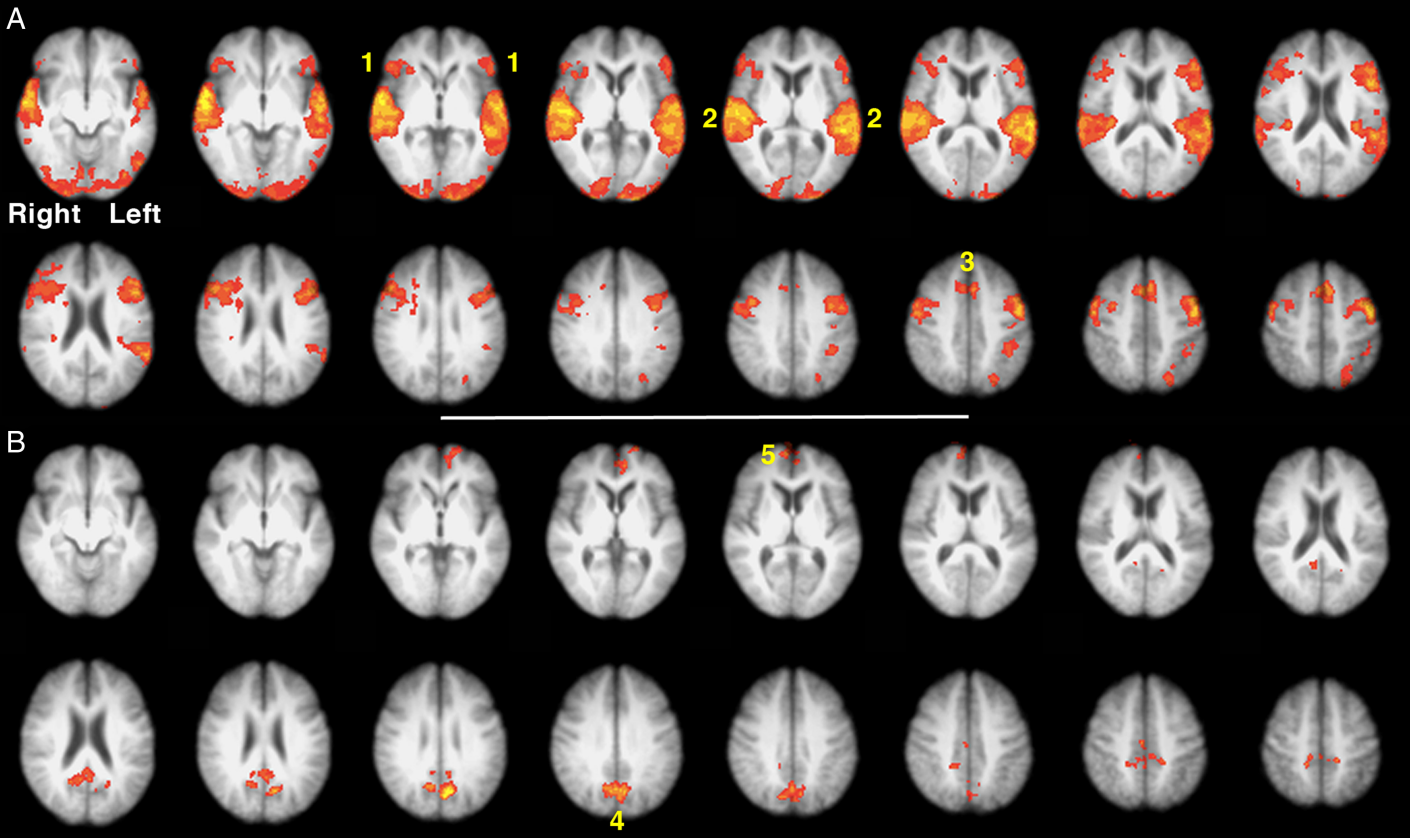
Two contrasts from Study 2: all trials with correct responses > all trials with incorrect responses (Panel *A*), and vice versa (Panel *B*), voxel-level threshold *Z* > 2.3, cluster-level threshold *P* < 0.05. (1) Anterior insula and adjacent frontal operculum (aI/Fop), (2) bilateral superior temporal gyri (STG), (3) dorsal anterior cingulate cortex and adjacent superior frontal gyrus (dACC/SFG), (4) precuneus and adjacent posterior cingulate cortex, (5) ventral medial prefrontal cortex.

Response trials from Study 2 were separated into correct and incorrect across all Listening conditions. Direct contrasts between these 2 groups of scan data for the preceding listening trials demonstrated that there was increased activity in the auditory cortices and the cingulo-opercular network when there was a subsequent correct response (Fig. 7*A*). In contrast, the reverse contrast demonstrated activity in anterior and posterior midline regions that are components of the DMN (Fig. 7*B*).

#### Summary of Findings from Study 2 Contrasted with Those from Study 1

The univariate contrast of (F_BABBLE_ + FM_DIOTIC_) with F_ALONE_ identified only a subset regions of activity observed in the first study: Activity was confined to the right aI/IFG, left PT/IPL, right posterolateral STG, and precuneus. On contrasting the dichotic (M_LEFT_F_RIGHT_ and F_LEFT_M_RIGHT_) with the diotic (FM_DIOTIC_) listening conditions, greater activity in the left PT/IPL and precuneus was associated with the spatial cues that assisted in the segregation of one speech stream from another. The conjunction of activity in the right aI/FOp in the first and second studies indicated a central role for this region in supporting speech stream segregation, independent of the context of task during listening (that is, a requirement for immediate or delayed recall of the content of the “attended” speech). In contrast, activity in the right MFG and SMG was strongly dependent on the context, most notably with the preparation for an immediate response to what was heard abolishing activity in the SMG. The ICA showed that this task was associated with a left fronto-temporoparietal network. Throughout all these networks, with the exception of SMG, activity was always greater during the Response trials relative to the Listening trials; but as in the first study, the widely distributed bilateral system comprising cingulo-opercular cortex, IFS/IPS, and cerebellar cortex was active during the listening trials, but was not modulated by speech-in-speech masking. Associated with activity in the cerebral and cerebellar hemispheres, Study 2 also demonstrated bilateral basal ganglia and thalamic activity, not observed in the first study. This can be attributed to the change in data acquisition for the second study (see Materials and Methods section) to improve sensitivity, which may have raised signal in the subcortical nuclei above the statistical threshold.

## Discussion

The 2 studies presented here were designed to demonstrate the local and distributed systems, particularly those involved in attention and cognitive control, when listening to a speaker and recalling what was said. Two experimental manipulations were designed to capture everyday listening conditions: the presence or absence of background competing speech; and task demand, namely delayed or immediate recall of what had been said by the “attended” speaker. Based on the results of the 2 fMRI studies, there were 3 cortical nodes that responded to speech-in-speech masking irrespective of the task demand: the precuneus, the left PT/IPL, and the right aI/FOp. We will start by discussing these common regions.

As lesions of the precuneus are extremely rare, neuropsychological lesion-deficit analyses to determine the function of this region do not exist. However, functional neuroimaging studies have implicated this region in a number of quite different functions (Cavanna and Trimble 2006). This must relate to multiple overlapping components within this region that form parts of anatomically and functionally dissociable networks, as has been shown for the adjacent posterior cingulate cortex (Leech and Sharp 2014). One function of the precuneus is egocentric spatial orientation, which has often been considered in terms of visuospatial navigation (for review, see Boccia et al. 2014). The precuneus is a component of the “dorsal attentional network” (DAN), which incorporates the dorsal precuneus and bilateral medial intraparietal sulci, superior parietal lobules, midline supplementary eye field, and frontal eye fields. The DAN has been most often investigated in relation to its response to visual tasks, becoming active as participants voluntarily focus attention on perceptually distinctive visual stimuli that are salient within the context of a specific task-dependent goal (for reviews, see Corbetta et al. 2008; Corbetta and Shulman 2011). However, one recent study has also strongly implicated the precuneus in detecting a target sound in complex acoustic environments (Zündorf et al. 2013). In the present study, this region was more active during the diotic presentation of 2 speakers compared with attending to a single speaker, and therefore in the absence of separate spatial cues for the 2 speakers. This is compatible with a top-down role in the detection of the salient speech stream based on nonspatial perceptual cues, such as voice pitch; but, as in the study of Zündorf et al. (2013), activity increased significantly when there were auditory cues indicating a spatial separation of the 2 speakers. Furthermore, associated with this increased activity with spatial cues, there was also an increase in activity in regions located to the supplementary eye field and the frontal eye fields. Although this was unexpected, and therefore appropriate recordings were not made, in future studies, it will of interest to determine whether spatial cues during speech stream segregation are accompanied by automatic eye movements toward the “attended” speaker.

Although of different design, and employing nonverbal auditory stimuli, the study of Zündorf et al. (2013) also demonstrated an increased response of the left PT to spatial cues, with evidence of some right posterior temporal involvement. Across the 2 studies reported here, activity in the left PT increased in response to one speaker, increased further when there was more than one speaker, and was greatest when spatial cues were present. Griffiths and Warren (2002) proposed that the PT is a computational hub, directing both spectrotemporal and spatial information to wider distributed networks engaged in the identification, semantic recognition, and auditory stream segregation of sounds, both environmental and verbal. These authors specifically proposed that the PT may be a central node in solving the “cocktail party” problem, and the results presented here support this hypothesis. Interestingly, a clinical study on stroke patients by Zündorf et al. (2014), and using the complex nonverbal sounds used in their earlier study (Zündorf et al. 2013), indicated that the right posterior temporal cortex, including the PT, is central to sound localization. However, patients with lesions that included the left PT were underrepresented because such patients were often too language-impaired to participate. We would argue on the basis of the present study that segregating one speech stream from others, using all available nonspatial and spatial cues, is dependent on the left PT, although activity also evident in the right posterior STG suggests that this function may be shared between the cerebral hemispheres.

We turn now to the role of the right aI/FOp. A decade ago, it was proposed that this region is specialized for initiating response inhibition and task switching (reviewed in Aron et al. 2014). More recently, Hampshire et al. (2010) demonstrated that this region, as part of the cingulo-opercular network, becomes active during the detection of important cues, irrespective of whether this is followed by inhibition or generation of a motor response, or even no external response at all. In their study, across a range of tasks that resulted in activity in both the cingulo-opercular and the bilateral IFS/IPS networks, activity was preferentially greater in the cingulo-opercular network for tasks that most depended on working memory. In the model proposed by Menon and Uddin (2010), the aI/FOp, forming a component of the cingulo-opercular network, is a core node involved in the generation of control signals following the perception of salient environmental events. These signals direct attentional, working memory and other higher-order control systems toward the mental processing of these events. Efficient processing of external stimuli depends on deactivation of the DMN (Kelly et al. 2008), a system considered to direct mental processing toward stimulus-independent internal thoughts and ruminations, with its functional connectivity increasing during brain development (Fair et al. 2008). Breakdown of this connectivity as a consequence of diffuse white matter injury following TBI has been shown to result in an association between impaired attention and a failure to deactivate the DMN (Bonnelle et al. 2011). More specifically, this failure efficiently to deactivate the DMN following TBI has been related to the tract connecting the right aI/FOp and dACC (Bonnelle et al. 2012), a consequence of which was impaired performance on a speeded visual task. This result was reflected in the analysis of the error trials in Study 2, which demonstrated that listening trials associated with a subsequent incorrect response was associated with a failure to deactivate the DMN relative to the trials followed by a correct response. This effect was presumably the neural signature of momentary lapses of attention to the stimuli (Weissman et al. 2006).

The most evident difference between the 2 studies was the functional dissociations between the response of the right dorsolateral prefrontal cortex, centered on the (MFG) and inferior parietal cortex (SMG). In the first study, activity within the ventral right frontoparietal network was only associated with the presence of a competing speaker. Therefore, the linguistic and semantic processing of heard speech, and the encoding of the information as episodic memories in response to the task demand of remembering the information until the end of the scanning session, required minimal involvement of this system. However, perceptual difficulty as the result of the presence of a competing speaker markedly increased activity in both the frontal and parietal components. One explanation for this is the need for an increase in sustained attention when attempting to encode the information conveyed by the “attended” speaker on the perceptually difficult trials, and this system has been associated with sustained attention (for a review, see Singh-Curry and Husain 2009). Changing the task demands in Study 2 so that an immediate response to what had been heard was required abolished activity in the right SMG, irrespective of perceptual difficulty, while resulting in increased activity in the right MFG across all trials. Therefore, a task demand with reliance on working memory rather than longer-term episodic memory encoding meant that this ventral right frontoparietal system was no longer influenced by the need for speech stream segregation. The ICA analysis demonstrated that one component (Component 4), as well as demonstrating activity in cingulo-opercular and IFS/IPS networks, revealed correlated activity in the left IFG, posterior inferolateral temporal lobe, and inferior parietal cortex. This would indicate the operation of a left hemisphere verbal working memory system, which was also shown to be active during the Response trials. Therefore, the task demand had a major influence on ventral right and left parietal networks, with tasks depending heavily on working memory resulting exclusively in left hemisphere involvement, whereas episodic memory encoding when increased attention was required because of perceptual difficulty depended on right hemisphere involvement.

The cingulo-opercular and IFDS/IPS networks are domain-general systems for cognitive control and attention and are active across many different kinds of task (Hampshire et al. 2012; Fedorenko et al. 2013). It was not surprising that they were most active during the Response trials of Study 2, although the ICA analyses of both studies demonstrated that they were also active during the attentive demands of the listening trials. However, activity in these systems was not modulated by the perceptual difficulty associated with speech-in-speech masking. The one exception, as previously discussed, was the right aI/FOp component of the cingulo-opercular network, strongly influenced by speech-in-masking, which indicates a particular role for this region in regulating attention and cognitive control as perceptual difficulty increases.

To summarize, the 2 studies described here have demonstrated the role of networks active during speech stream segregation during attentive listening, and whether their degree of involvement is influenced by the duration over which the verbal information conveyed over time has to be held in memory. Three regions, in particular, were central to speech stream segregation: the left PT, precuneus, and right aI/FOp. Focal lesions of the precuneus are rare, but patients with a stroke affecting the right aI/FOp are presumably not that uncommon. Therefore, a future lesion-deficit analysis could be performed to confirm the proposal that the right aI/FOp is central to activating attention and memory systems, and deactivating the DMN, when listening to a speaker in a “cocktail party” auditory environment. Our result supports the hypothesis that impaired speech perception and comprehension following an aphasic stroke result in increased reliance on the function of the right aI/FOp (Geranmayeh et al. 2014).

## Supplementary Material

Supplementary material can be found at: http://www.cercor.oxfordjournals.org/.

## Funding

This work was supported by the Medical Research Council (G1100423). Funding to pay the Open Access publication charges for this article was provided by the Medical Research Council.

## Supplementary Material

Supplementary Data

## References

[BHU325C1] Algazi VR Avendano C Duda RO 2001 Elevation localization and head-related transfer function analysis at low frequencies. J Acoust Soc Am. 109:1110–1122. 11303925 10.1121/1.1349185

[BHU325C2] Aron AR Robbins TW Poldrack RA 2014 Inhibition and the right inferior frontal cortex: one decade on. Trends Cogn Sci. 18:177–185. 24440116 10.1016/j.tics.2013.12.003

[BHU325C3] Beckmann CF DeLuca M Devlin JT Smith SM 2005 Investigations into resting-state connectivity using independent component analysis. Philos Trans R Soc Lond B Biol Sci. 260:1001–1013. 16087444 10.1098/rstb.2005.1634 PMC1854918

[BHU325C4] Beckmann CF Jenkinson M Smith SM 2003 General multilevel linear modelling for group analysis in FMRI. Neuroimage. 20:1052–1063. 14568475 10.1016/S1053-8119(03)00435-X

[BHU325C5] Beckmann CF Smith SM 2004 Probabilistic independent component analysis for functional magnetic resonance imaging. IEEE Trans Med Imaging. 23:137–152. 14964560 10.1109/TMI.2003.822821

[BHU325C6] Beckmann CF Smith SM 2005 Tensorial extensions of independent component analysis for multisubject fMRI analysis. Neuroimage. 25:294–311. 15734364 10.1016/j.neuroimage.2004.10.043

[BHU325C7] Bennett CM Miller MB 2010 How reliable are the results from functional magnetic resonance imaging? Ann N Y Acad Sci. 1191:133–155. 20392279 10.1111/j.1749-6632.2010.05446.x

[BHU325C8] Boccia M Nemmi F Guariglia C 2014 Neuropsychology of environmental navigation in humans: review and meta-analysis of fMRI studies in healthy participants. Neuropsychol Rev. 24:236–251. 24488500 10.1007/s11065-014-9247-8 PMC4010721

[BHU325C9] Bonnelle V Ham TE Leech R Kinnunen KM Mehta MA Greenwood RJ Sharp DJ 2012 Salience network integrity predicts default mode network function after traumatic brain injury. Proc Nactl Acad Sci USA. 109:4690–4695. 10.1073/pnas.1113455109 PMC3311356 22393019

[BHU325C10] Bonnelle V Leech R Kinnunen KM Ham TE Beckmann CF De Boissezon X Greenwood RJ Sharp DJ 2011 Default mode network connectivity predicts sustained attention deficits after traumatic brain injury. J Neurosci. 31:13442–13451. 21940437 10.1523/JNEUROSCI.1163-11.2011 PMC6623308

[BHU325C11] Brainard DH 1997 The psychophysics toolbox. Spat Vis. 10:433–436. 9176952

[BHU325C12] Bregman AS 1990 Auditory Scene Analysis: The Perceptual Organization of Sound. Cambridge, MA: MIT Press.

[BHU325C13] Brungart DS 2001 Informational and energetic masking effect in the perception of two simultaneous talkers. J Acoust Soc Am. 109:1101–1109. 11303924 10.1121/1.1345696

[BHU325C14] Carlyon RP 2004 How the brain separates sounds. Trends Cogn Sci. 8:465–471. 15450511 10.1016/j.tics.2004.08.008

[BHU325C15] Cavanna AE Trimble MR 2006 The precuneus: a review of its functional anatomy and behavioural correlates. Brain. 129:564–583. 16399806 10.1093/brain/awl004

[BHU325C16] Corbetta M Patel G Shulman GL 2008 The reorienting system of the human brain: from environment to theory of mind. Neuron. 58:306–324. 18466742 10.1016/j.neuron.2008.04.017 PMC2441869

[BHU325C17] Corbetta M Shulman GL 2011 Spatial neglect and attention networks. Annu Rev Neurosci. 34:569–599. 21692662 10.1146/annurev-neuro-061010-113731 PMC3790661

[BHU325C18] Darwin CJ 2008 Listening to speech in the presence of other sounds. Philos Trans R Soc Lond B Biol Sci. 363:1011–1021. 17827106 10.1098/rstb.2007.2156 PMC2606793

[BHU325C19] Darwin CJ Hukin RW 2000a Effectiveness of spatial cues, prosody, and talker characteristics in selective attention. J Acoust Soc Am. 107:970–977. 10687706 10.1121/1.428278

[BHU325C20] Darwin CJ Hukin RW 2000b Effects of reverberation on spatial, prosodic, and vocal-tract size cues to selective attention. J Acoust Soc Am. 108:335–342. 10923896 10.1121/1.429468

[BHU325C21] Dosenbach NU Fair DA Cohen AL Schlaggar BL Petersen SE 2008 A dual-networks architecture of top-down control. Trends Cogn Sci. 12:99–105. 18262825 10.1016/j.tics.2008.01.001 PMC3632449

[BHU325C22] Dosenbach NU Fair DA Miezin FM Cohen AL Wenger KK Dosenbach RA Fox MD Snyder AZ Raichle ME Schlaggar BL 2007 Distinct brain networks for adaptive and stable task control in humans. Proc Natl Acad Sci USA. 104:11073–11078. 17576922 10.1073/pnas.0704320104 PMC1904171

[BHU325C23] Duncan J 2010 The multiple-demand (MD) system of the primate brain: mental programs for intelligent behaviour. Trends Cogn Sci. 14:172–179. 20171926 10.1016/j.tics.2010.01.004

[BHU325C24] Duncan J 2013 The structure of cognition: attentional episodes in mind and brain. Neuron. 80:35–50. 24094101 10.1016/j.neuron.2013.09.015 PMC3791406

[BHU325C25] Fair DA Cohen AL Dosenbach NU Church JA Miezin FM Barch DM Raichle ME Petersen SE Schlaggar BL 2008 The maturing architecture of the brain's default network. Proc Natl Acad Sci USA. 105:4028–4032. 18322013 10.1073/pnas.0800376105 PMC2268790

[BHU325C26] Fedorenko E Duncan J Kanwisher N 2013 Broad domain generality in focal regions of frontal and parietal cortex. Proc Natl Acad Sci USA. 110:16616–16621. 24062451 10.1073/pnas.1315235110 PMC3799302

[BHU325C27] Feng AS Ratnam R 2000 Neural basis of hearing in real-world situations. Annu Rev Psychol. 51:699–725. 10751985 10.1146/annurev.psych.51.1.699

[BHU325C28] Fox MD Snyder AZ Vincent JL Corbetta M Van Essen DC Raichle ME 2005 The human brain is intrinsically organized into dynamic, anticorrelated functional networks. Proc Natl Acad Sci USA. 102:9673–9678. 15976020 10.1073/pnas.0504136102 PMC1157105

[BHU325C29] Geranmayeh F Brownsett SL Wise RJ 2014 Task-induced brain activity in aphasic stroke patients: what is driving recovery? Brain. 137:2632–2648. 24974382 10.1093/brain/awu163 PMC4163030

[BHU325C30] Griffiths TD Warren JD 2002 The planum temporale as a computational hub. Trends Neurosci. 27:348–353. 12079762 10.1016/s0166-2236(02)02191-4

[BHU325C31] Hall DA Haggard MP Akeroyd MA Palmer AR Summerfield AQ Elliott MR Gurney EM Bowtell RW 1999 “Sparse” temporal sampling in auditory fMRI. Hum Brain Mapp. 7:213–223. 10194620 10.1002/(SICI)1097-0193(1999)7:3<213::AID-HBM5>3.0.CO;2-N PMC6873323

[BHU325C32] Hampshire A Chamberlain SR Monti MM Duncan J Owen AM 2010 The role of the right inferior frontal gyrus: inhibition and attentional control. Neuroimage. 50:1313–1319. 20056157 10.1016/j.neuroimage.2009.12.109 PMC2845804

[BHU325C33] Hampshire A Highfield RR Parkin BL Owen AM 2012 Fractionating human intelligence. Neuron. 76:1225–1237. 23259956 10.1016/j.neuron.2012.06.022

[BHU325C34] Hyvärinen A 1999 Fast and robust fixed-point algorithms for independent component analysis. IEEE Trans Neural Netw. 10:626–634. 18252563 10.1109/72.761722

[BHU325C35] Jenkinson M Bannister P Brady M Smith S 2002 Improved optimization for the robust and accurate linear registration and motion correction of brain images. Neuroimage. 17:825–841. 12377157 10.1016/s1053-8119(02)91132-8

[BHU325C36] Kalikow DN Stevens KN Elliott LL 1977 Development of a test of speech intelligibility in noise using sentence materials with controlled word predictability. J Acoust Soc Am. 61:1337–1351. 881487 10.1121/1.381436

[BHU325C37] Kelly AM Uddin LQ Biswal BB Castellanos FX Milham MP 2008 Competition between functional brain networks mediates behavioral variability. Neuroimage. 39:527–537. 17919929 10.1016/j.neuroimage.2007.08.008

[BHU325C38] Klinkenberg I Sambeth A Blokland A 2011 Acetycholine and attention. Behav Brain Res. 221:430–442. 21108972 10.1016/j.bbr.2010.11.033

[BHU325C39] Leech R Braga R Sharp DJ 2012 Echoes of the brain within the posterior cingulate cortex. J Neurosci. 32:215–222. 22219283 10.1523/JNEUROSCI.3689-11.2012 PMC6621313

[BHU325C40] Leech R Kamourieh S Beckmann CF Sharp DJ 2011 Fractionating the default mode network: distinct contributions of the ventral and dorsal posterior cingulate cortex to cognitive control. J Neurosci. 31:3217–3224. 21368033 10.1523/JNEUROSCI.5626-10.2011 PMC6623935

[BHU325C41] Leech R Sharp DJ 2014 The role of the posterior cingulate cortex in cognition and disease. Brain. 137:12–32. 23869106 10.1093/brain/awt162 PMC3891440

[BHU325C42] Menon V Uddin LQ 2010 Saliency, switching, attention and control: a network model of insula function. Brain Struct Funct. 214:655–667. 20512370 10.1007/s00429-010-0262-0 PMC2899886

[BHU325C43] Obleser J Wise RJ Alex Dresner M Scott SK 2007 Functional integration across brain regions improves speech perception under adverse listening conditions. J Neurosci. 27:2283–2289. 17329425 10.1523/JNEUROSCI.4663-06.2007 PMC6673469

[BHU325C44] Robertson IH 2014 A right hemisphere role in cognitive reserve. Neurobiol Aging. 35:1375–1385. 24378088 10.1016/j.neurobiolaging.2013.11.028

[BHU325C45] Roca M Parr A Thompson R Woolgar A Torralva T Antoun N Manes F Duncan J 2010 Executive function and fluid intelligence after frontal lobe lesions. Brain. 133:234–247. 19903732 10.1093/brain/awp269 PMC2801324

[BHU325C46] Schwarzbauer C Davis MH Rodd JM Johnsrude I 2006 Interleaved silent steady state (ISSS) imaging: a new sparse imaging method applied to auditory fMRI. Neuroimage. 29:774–782. 16226896 10.1016/j.neuroimage.2005.08.025

[BHU325C47] Shallice T Stuss DT Picton TW Alexander MP Gillingham S 2008 Mapping task switching in frontal cortex through neuropsychological group studies. Front Neurosci. 2:79–85. 18982110 10.3389/neuro.01.013.2008 PMC2570079

[BHU325C48] Singh-Curry V Husain M 2009 The functional role of the inferior parietal lobe in the dorsal and ventral stream dichotomy. Neuropsychologia. 47:1434–1448. 19138694 10.1016/j.neuropsychologia.2008.11.033 PMC2697316

[BHU325C49] Smith SM 2002 Fast robust automated brain extraction. Hum Brain Mapp. 17:143–155. 12391568 10.1002/hbm.10062 PMC6871816

[BHU325C50] Smith SM Fox PT Miller KL Glahn DC Fox PM Mackay CE Filippini N Watkins KE Toro R Laird AR 2009 Correspondence of the brain's functional architecture during activation and rest. Proc Natl Acad Sci USA. 106:13040–13045. 19620724 10.1073/pnas.0905267106 PMC2722273

[BHU325C51] Smith SM Jenkinson M Woolrich MW Beckmann CF Behrens TE Johansen-Berg H Bannister PR De Luca M Drobnjak I Flitney DE 2004 Advances in functional and structural MR image analysis and implementation as FSL. Neuroimage. 23 (Suppl 1):S208–S219. 15501092 10.1016/j.neuroimage.2004.07.051

[BHU325C52] Snyder JS Alain C 2007 Toward a neurophysiological theory of auditory stream segregation. Psychol Bull. 133:780–799. 17723030 10.1037/0033-2909.133.5.780

[BHU325C54] Vincent JL Kahn I Snyder AZ Raichle ME Buckner RL 2008 Evidence for a frontoparietal control system revealed by intrinsic functional connectivity. J Neurophysiol. 100:3328–3342. 18799601 10.1152/jn.90355.2008 PMC2604839

[BHU325C55] Weissman DH Roberts KC Visscher KM Woldorff MG 2006 The neural bases of momentary lapses in attention. Nat Neurosci. 9:971–978. 16767087 10.1038/nn1727

[BHU325C56] Woolgar A Bor D Duncan J 2013 Global increase in task-related fronto-parietal activity after focal frontal lobe lesion. J Cogn Neurosci. 25:1542–1552. 23767925 10.1162/jocn_a_00432

[BHU325C57] Woolgar A Hampshire A Thompson R Duncan J 2011 Adaptive coding of task-relevant information in human frontoparietal cortex. J Neurosci. 31:14592–14599. 21994375 10.1523/JNEUROSCI.2616-11.2011 PMC6703398

[BHU325C58] Zündorf IC Karnath HO Lewald J 2014 The effect of brain lesions on sound localization in complex acoustic evnironments. Brain. 137:1410–1418. 24618271 10.1093/brain/awu044

[BHU325C59] Zündorf IC Lewald J Karnath HO 2013 Neural correlates of sound localization in complex acoustic environments. PLoS One. 8:e64259. 23691185 10.1371/journal.pone.0064259 PMC3653868

