# Supplementary material for: Neural Systems Involved When Attending to a Speaker
Source: Cereb Cortex. 2015 Jan 16;25(11):4284–98. doi: 10.1093/cercor/bhu325 (PMC4816781; doi:10.1093/cercor/bhu325)
Supplement: Supplementary Data [file supp_25_11_4284__index.html]

Neural Systems Involved When Attending to a Speaker — Neural Systems Involved When Attending to a Speaker — Supplementary Data 

# Neural Systems Involved When Attending to a Speaker

## Supplementary Data

Supplementary Data

**Files in this Data Supplement:**

- Supplementary Table 1 - doc file
