# Supplementary material for: Neural Systems Involved When Attending to a Speaker
Source: Cereb Cortex. 2015 Jan 16;25(11):4284–98. doi: 10.1093/cercor/bhu325 (PMC4816781; doi:10.1093/cercor/bhu325)
Supplement: Supplementary Data [file supp_bhu325_bhu325supp_table1.doc]

Table S1 – ‘Centre of mass’ coordinates for significant clusters in the multivariate analysis of Studies 1 and 2. x, y and z are in MNI space. IFS = inferior frontal sulcus; IPS = intra-parietal sulcus; STG = superior temporal gyrus; dACC/SFG = dorsal anterior cingulate cortex/superior frontal gyrus; aI/FOp = anterior insular cortices/frontal opercula; MFG/SMG = middle frontal gyrus/supramarginal gyrus.

| Contrasts | Hem | Region Label | x | y | z |
| --- | --- | --- | --- | --- | --- |
| **Study 1** |  |  |  |  |  |
| All Listening conditions > Silence |  |  |  |  |  |
|  | Left | STG | -54 | -26 | 0 |
|  | Right | STG | 62 | -18 | -4 |
|  |  |  |  |  |  |
| Speech > Silence |  |  |  |  |  |
|  | Left | IFS | -30 | 10 | 44 |
|  | Left | IPS | -34 | -62 | 48 |
|  | Left | Lateral cerebellar hemisphere | -30 | -70 | -32 |
|  | Left | STG | -58 | -26 | -4 |
|  | Right | IFS | 46 | 10 | 40 |
|  | Right | IPS | 38 | -58 | 44 |
|  | Right | Lateral cerebellar hemisphere | 26 | -74 | -40 |
|  | Right | STG | 62 | -18 | -4 |
|  |  | dACC/SFG | -2 | 18 | 52 |
|  |  |  |  |  |  |
| Diotic speech > Single |  |  |  |  |  |
|  | Left | STG | -58 | -22 | 0 |
|  | Right | aI/FOp | 38 | 26 | -4 |
|  | Right | STG | 58 | -38 | 12 |
|  |  | dACC/SFG | 6 | 18 | 44 |
|  |  | MFG/SMG | 50 | 18 | 3 |
|  |  | Precuneus | 10 | -74 | 40 |
|  |  |  |  |  |  |
| **Study 2** |  |  |  |  |  |
| Response > Listening |  |  |  |  |  |
|  | Left | aI/FOp | -30 | 26 | -4 |
|  | Left | IFS | -42 | 6 | 32 |
|  | Left | IPS | -22 | -70 | 52 |
|  | Left | STG | -50 | -26 | 8 |
|  | Left | Occipital cortex | -18 | -94 | 8 |
|  | Right | aI/FOp | 38 | 26 | -4 |
|  | Right | IFS | 46 | 6 | 32 |
|  | Right | IPS | 30 | -74 | 32 |
|  | Right | STG | 58 | -18 | 4 |
|  | Right | Occipital cortex | 26 | -90 | 16 |
|  |  | dACC/SFG | -2 | 14 | 52 |
|  |  |  |  |  |  |
| Response > All speech combined  All listening combined > Silence  F_ALONE_ > Silence |  |  |  |  |  |
|  | Left | aI/FOp | -42 | 30 | -4 |
|  | Left | IFS | -42 | 10 | 32 |
|  | Left | Inf parietal cortex | -58 | -50 | 24 |
|  | Left | IPS | -30 | -70 | 40 |
|  | Left | Visual cortex | -22 | -86 | -12 |
|  | Right | aI/FOp | 38 | 26 | -4 |
|  | Right | IFS | 42 | 10 | 32 |
|  | Right | IPS | 30 | -66 | 48 |
|  | Right | Visual cortex | 18 | -98 | 12 |
|  |  | ACC | -2 | 14 | 56 |
|  |  |  |  |  |  |
| Response > All listening speech combined  All listening combined > Silence  F_ALONE_ > Silence |  |  |  |  |  |
|  | Left | aI/Fop | -50 | 22 | -8 |
|  | Left | IFS | -46 | 18 | 24 |
|  | Left | IPS | -30 | -58 | 48 |
|  | Left | Lateral cerebellum | -26 | -82 | -20 |
|  | Right | aI/Fop | 50 | 26 | -12 |
|  | Right | IFS | 42 | 10 | 36 |
|  | Right | IPS | 30 | -62 | 56 |
|  | Right | Lateral cerebellum | 26 | -74 | -20 |
|  |  | ACC | -2 | 14 | 56 |
